# Safety Evaluation of *Bacillus subtilis* IDCC1101, Newly Isolated from Cheonggukjang, for Industrial Applications

**DOI:** 10.3390/microorganisms10122494

**Published:** 2022-12-16

**Authors:** Su-Hyeon Kim, Gashaw Assefa Yehuala, Won Yeong Bang, Jungwoo Yang, Young Hoon Jung, Mi-Kyung Park

**Affiliations:** 1 School of Food Science and Biotechnology, Kyungpook National University, Daegu 41566, Republic of Korea; 2 Food and Bio-Industry Research Institute, Kyungpook National University, Daegu 41566, Republic of Korea; 3 Department of Food Engineering, College of Biological and Chemical Engineering, Addis Ababa Science and Technology University, Addis Ababa 16417, Ethiopia; 4 Ildong Bioscience, Pyeongtaek-si 17957, Republic of Korea

**Keywords:** *Bacillus subtilis* IDCC1101, safety evaluation, industrial application, probiotics, genomic analysis

## Abstract

The present study aimed to evaluate the safety of *Bacillus subtilis* (BS) IDCC1101, newly isolated from Cheonggukjang in Korea. Genome sequencing of BS IDCC1101 was performed to investigate the presence of secondary metabolites, virulence, antibiotic resistance, and mobile elements. Its phenotypic safety analyses included antibiotic susceptibility, enzyme activity, carbohydrate utilization, production of biogenic amines (BAs) and D-/L-lactate, hemolytic activity, and toxicities in HaCaT cells and rats. The genome of BS IDCC1101 consisted of 4,118,950 bp with 3077 functional genes. Among them, antimicrobial and antifungal secondary metabolites were found, such as fengycin, bacillibactin, and bacilysin. Antibiotic resistance and virulence genes did not exhibit transferability since they did not overlap with mobile elements in the genome. BS IDCC1101 was susceptible to almost all antibiotics suggested for assessment of BS’s antibiotic susceptibility by EFSA guidelines, except for streptomycin. BS IDCC1101 showed the utilization of a wide range of 27 carbohydrates, as well as enzyme activities such as alkaline phosphatase, esterase, esterase lipase, naphthol-AS-BI-phosphohydrolase, α-galactosidase, β-galactosidase, α-glucosidase, and β-glucosidase activities. Additionally, BS IDCC1101 did not exhibit the production of D-/L-lactate and hemolytic activities. Its toxicity in HaCaT cells and rats was also not detected. Thus, these genotypic and phenotypic findings indicate that BS IDCC1101 can be safely used for industrial applications.

## 1. Introduction

The global market for probiotics has been growing rapidly and continuously, and its global market is estimated at USD 48.8 billion in 2019 and is expected to reach USD 94.5 billion by 2027 [1]. Probiotics, health-improving microorganisms, have been used in various food products and proposed as a food supplement or therapy for several infectious diseases due to their health benefits [2]. Probiotics administered to humans improve the balance of microbial communities, intestinal regulation, and the immune system [2,3,4,5]. Among the probiotic strains, some strains of *Bacillus* spp. have begun to be used for industrial applications, such as probiotic dietary supplements for humans and animal feed [6].

*Bacillus*, in particular, is a Gram-positive bacterium with a long history of use in the traditional food industry in many Asian countries, such as Japanese natto [7], Korean kimchi [8], Egyptian kishk [9], and Thai thua nao [10]. It has expanded to more than 100 species (https://www.dsmz.de/search, accessed on 1 October 2022) over the past half-century. However, only a few of these species have been used as probiotics for humans, including *subtilis*, *licheniformis*, *clausii*, *coagulans*, *cereus*, *pumilus*, and *laterosporus*, due to the existence of transmissible antibiotic-resistant determinants and the production of enterotoxins and/or emetic toxins in some *Bacillus* strains [11]. Thus, the industrial use of *Bacillus* spp. requires strict standards for ensuring safety. The USA’s Food and Drug Administration (FDA), Health Canada, and the European Food Safety Authority (EFSA) have introduced legislation on this matter despite the absence of an international standard for safety evaluation [12]. 

*Bacillus subtilis* is increasingly attracting attention as a promising probiotic due to its growth versatility and nutrient utilization, high level of enzyme production, antimicrobial substance production, sporulation, and easy cultivation [13]. In addition, it has an excellent stability profile in unfavorable conditions such as gastric acid, bile salts, and high heat [14]. In fact, *B. subtilis* is considered a group in the EFSA-qualified presumption of safety (QPS) list for its industrial and commercial applications [15]. Meanwhile, more studies have reported the possible presence of toxins in several strains of the *B. subtilis* group including emetic toxins, hemolytic and non-hemolytic enterotoxins, and the possibility of transmissible antibiotic resistance genes [16,17,18,19,20,21,22]. However, information concerning these issues is limited for the newly identified *B*. *subtilis*. Thus, assessing novel *B*. *subtilis* strains is inevitable before their industrial use to ensure safety and consumer confidence (EFSA, 2005).

This study investigated the safety and biochemical properties of *Bacillus subtilis* IDCC 1101 isolated from Cheonggukjang, a traditional Korean fermented soybean food, using the guidelines recommended by the FDA [12]. A whole-genome sequence analysis was used to assess the safety of *B. subtilis* IDCC1101 and determine the presence of virulence and antibiotic resistance genes and mobile genetic elements. Furthermore, to ensure that *B. subtilis* IDCC1101 can be commercially used in the food and pharmaceutical industries, it was examined for antibiotic susceptibility, enzyme activity, carbohydrate utilization, biogenic amine (BA) production, D-/L-lactate formation, cytotoxicity, and acute oral toxicity.

## 2. Materials and Methods

### 2.1. Bacterial Strains and Culture Conditions

*Bacillus subtilis* (BS) IDCC1101 was isolated from Cheonggukjang and obtained from Ildong Bioscience Co. (Gyeonggi-do, Korea). BS IDCC1101 was aerobically cultured in Luria-Bertani (LB) broth (Difco Laboratories Inc., Sparks, MD, USA) at 37 °C for 24 h. After centrifugation at 6000× *g* for 4 min and washing three times with phosphate buffered saline (PBS, pH 7.4, Life Technologies Ltd., Paisley, UK), the bacterial pellet was resuspended into PBS. Consequently, the concentration of the bacterial suspension was adjusted to 8 log CFU/mL by measuring the optical density at 640 nm.

### 2.2. Genome Sequencing and Taxonomic Classification of BS IDCC1101

BS IDCC1101 genomic DNA was extracted using a Maxwell^®^ 16 LEV Blood DNA Kit and a Maxwell^®^ 16 Buccal Swab LEV DNA Purification Kit (Promega Co., Madison, WI, USA) according to the manufacturer’s instructions. Whole-genome sequencing was performed at Macrogen Inc. (Seoul, Korea) using a PacBio RS II instrument (Pacific Biosciences of California, Inc., Menlo Park, CA, USA) with an Illumina platform (Illumina Inc., San Diego, CA, USA). The raw sequence reads were trimmed using Trimmomatic [23] and then de novo assembled using the RS Hierarchical Genome Assembly Process (v.3.0). After annotating the open reading frames (ORFs) of the assembled sequence using Prokka (v1.13), Rapid Annotations using Subsystems Technology (RAST) server [24], and BLASTP, the ORFs were grouped with EggNOG functional categorization based on non-supervised clustering of orthologous groups [25]. Finally, the genome map of BS IDCC1101 was constructed using a BLAST ring image generator [26].

The complete genome of BS IDCC1101 was screened using BLASTN, and the average nucleotide identity (ANI) value with the most similar *B*. *subtilis* strains was calculated using OrthoANI Tool v0.93.1 [27]. Additionally, six housekeeping genes of BS IDCC1101 and 15 other *Bacillus* genomes were procured from the National Center for Biotechnology Information (NCBI) database to identify its taxonomic classification. The standard housekeeping genes for the *Bacillus* spp. included *groEL* (gene ID: 938045), *gyrA* (gene ID: 940002), *polC* (gene ID: 939620), *purH* (gene ID: 936053), *rpoB* (geneID: 936335), and 16S rRNA (gene ID: 936895) derived from type strain 168, which is one of the most well-studied strains of *B*. *subtilis* [7]. The six genes of each strain were aligned and concatenated into a single sequence. Subsequently, the concatenated sequences were multi-aligned by MAFFT [28] (https://mafft.cbrc.jp/alignment/server/, accessed on 20 October 2022). A phylogenetic analysis was performed using the neighbor-joining method with 1000 bootstrap values via MEGA X [29].

### 2.3. In Silico Identification of Secondary Metabolites via antiSMASH

The BS IDCC1101 genome was compared with the antiSMASH bacterial version (https://antismash.secondarymetabolites.org/#!/start, v.6.0.1, accessed on 25 October 2022) to assess its ability to produce secondary metabolites [30]. The criteria for the analysis included KnownClusterBlast, ActiveSiteFinder, RREFinder, and SubClusterBlast under the relaxed strictness version.

### 2.4. Safety Assessment of BS IDCC1101

The safety of BS IDCC1101 was evaluated by confirming the presence of virulence factors in the genome. The BS IDCC1101 genome was compared with the sequences of virulence factors or proteins involved in their synthesis curated from the virulence factor database (VFDB) [31] with thresholds of >80% identity and >70% coverage.

The enterotoxin and emetic toxin genes were screened in BS IDCC by PCR amplification of its gDNA using primer sets designed by previous studies [32,33]. The primer sequences are listed in Table 1. The gDNA sample was prepared by preheating at 95 °C for 5 min and then amplified using Incline^TM^ *Taq* polymerases (Inclone Biotech, Daejeon, Korea) and a T-100 Thermocycler (Bio-Rad Laboratories Inc., Hercules, CA, USA). The PCR reaction was conducted by 30 cycles of denaturation at 95 °C for 1 min, annealing at 57 °C for 30 s, and extension at 72 °C for 1 min. The PCR products were detected on 1.5% agarose gel stained with ethidium bromide and observed under UV. *B*. *cereus* ATCC 14,579 was used as a positive control.

### 2.5. Hemolysis Activity of BS IDCC1101

The hemolytic activity of BS IDCC1101 was determined following the method described in a previous study (Halder et al., 2017). Briefly, the overnight cultured BS IDCC1101 in LB was streaked on a blood agar plate (Difco Laboratories Inc., Sparks, MD, USA) and incubated at 37 °C for 72 h. *Staphylococcus aureus* ATCC 25923 was used as a positive control. Depending on the characteristics of the zone formed around the colonies, it can be distinguished as α-hemolysis (deep green zone), β-hemolysis (clear zone), or γ-hemolysis (no zone) [34].

### 2.6. Genotypic and Phenotypic Antibiotic Susceptibility of BS IDCC1101

For genomic analysis of BS IDCC1101, the antibiotic resistance genes in the BS IDCC1101 genome were screened using the ResFinder database (v. 4.1) [35] with thresholds of >80% identity and >60% coverage. After that, the mobile genetic elements in the genome were searched to determine the possibility of horizontal gene transfer. The mobile genetic elements, including transposases and plasmids, and prophage regions were identified using the BLASTP algorithm and the PHASTER database [36], respectively.

The phenotypic antibiotic susceptibility of BS IDCC1101 was determined against nine antibiotic substances recommended by EFSA, such as ampicillin, vancomycin, gentamicin, kanamycin, streptomycin, erythromycin, clindamycin, tetracycline, and chloramphenicol (Sigma-Aldrich Co., St. Louis, MO, USA), according to the Clinical Laboratory Standards Institute (CLSI) guidelines. A bacterial suspension of BS IDCC1101 (5 × 10^5^ CFU/mL) and each antibiotic with various concentrations of 0.125–1024 µg/mL were mixed in a 96-well microplate and anaerobically incubated at 37 °C for 18 h. The optical density of the mixture was measured using a microplate reader (Agilent Technologies Inc., Winooski, VT, USA), and the minimum inhibition concentrations (MICs) were determined as the lowest concentrations that completely inhibited cell growth. Antibiotic susceptibility of BS IDCC1101 was assessed based on the cutoff values reported within the EFSA guidelines [37].

### 2.7. Enzyme Activities and Carbohydrate Utilization of BS IDCC1101

The enzyme activities of BS IDCC1101 were determined using an API ZYM KIT (bioMérieux Inc., Marcy l’Etoile, France) according to the manufacturer’s instructions. The bacterial suspension of BS IDCC1101 (9 log CFU/mL) was inoculated into a well plate of the kit, and the plate was incubated at 37 °C for 4 h. One drop of ZYM A and ZYM B reagents was added to each well prior to incubation for 5 min at RT. Color changes of the mixture were observed to determine its enzyme activity. 

Carbohydrate utilization of BS IDCC1101 was assessed using an API 50 CHL/CHB KIT (bioMérieux Inc.). The overnight culture of BS IDCC1101 was centrifugated at 6000 rpm for 8 min at 4 °C, and the bacterial pellet (6.0 × 10^8^ CFU/mL) was resuspended into API 50 API 50 CHL/CHB medium. The resuspended bacterial suspension was then inoculated into each well plate of the kit containing 49 different carbohydrates. After incubation at 37 °C for 48 h, the color changes were observed.

### 2.8. Biogenic Amines Production of BS IDCC1101

Biogenic amine (BA) production by BS IDCC1101 was determined per a previously described method [38] with minor modifications. Cell free supernatant (CFS) was obtained by centrifugation of the overnight culture at 6000 rpm for 30 min at 4 °C followed by filtration through a 0.22 µm membrane. To extract BAs, 0.5 mL of CFS was mixed with 0.5 mL of 0.1 M HCl and filtered through a 0.45 µm membrane. For derivatization, 1 mL of the extracted BAs was incubated in a water bath at 70 °C for 10 min, followed by the addition of 200 µL of saturated NaHCO_3_, 20 µL of 2 M NaOH, and 0.5 mL of dansyl chloride (10 mg/mL in acetone). The derivatized BAs were subsequently mixed with 200 µL of L-proline (100 mg/mL in H_2_O) and incubated in the dark at 22 °C for 15 min. Acetonitrile (high-performance liquid chromatography [HPLC] grade; Sigma-Aldrich, St. Louis, MO, USA) was added to reach a final volume of 5 mL. Finally, the BAs were quantified via HPLC (Agilent 1260, Agilent Technologies, Santa Clara, CA, USA) using a Cl8 column (YMC-Triart, 4.6 × 250 mm, YMC, Kyoto, Japan). An aqueous acetonitrile solution (67:33 of H_2_O, *v*/*v*) was used as a mobile phase at a constant flow rate of 0.8 mL/min. Peaks were detected at 254 nm using a UV detector (G7115A, Agilent Technologies, Santa Clara, CA, USA) and quantified according to each calibration curve for tyramine, histamine, putrescine, 2-phenethylamine, cadaverine, and tryptamine (Sigma-Aldrich, St. Louis, MO, USA).

### 2.9. Proportion of D-/L-Lactate of BS IDCC1101

The D-/L- lactate levels of BS IDCC1101 were measured using a D-/L-Lactate Rapid Assay Kit (Megazyme, Bray, Ireland). An aliquot of 0.1 mL of CFS of BS IDCC1101 was mixed with the reagents provided by the kit. The absorbance of the mixture was measured at 340 nm, and the concentrations of D- and L-lactate were then calculated using the equations from the manufacturer’s protocol.

### 2.10. Evaluation of the Cytotoxicity of BS IDCC1101

To evaluate cytotoxicity in HaCaT cells, BS IDCC1101 was incubated in 10 mL of LB at 37 °C for 18 h. CFS from the culture was obtained via centrifugation at 6000 rpm for 30 min at 4 °C. HaCaT cells (2 × 10^5^ cell/mL) were cultured into a 96-well plate and allowed to reach 70–80% confluency. Each CFS and bacterial suspension of BS IDCC1101 (~7 log CFU/mL) was added to the HaCaT cells and incubated for 24 h at 37 °C. Subsequently, 10 µL 3-(4,5-dimethyl-2- thiazolyl)-2,5-diphenyl-2H-tetrazolium bromide (MTT) solution (5 mg/mL) (Sigma-Aldrich) was added to each well and further incubated for 4 h. The formazan crystals formed in HaCaT cells were dissolved and suspended in 100 µL of dimethyl sulfoxide (Sigma-Aldrich), and the optical density was measured using a microplate reader (Bio-Rad, Hercules, CA, USA) at 595 nm. Finally, its viability was expressed as a percentage of the initial density.

### 2.11. Acute Oral Toxicity Study of BS IDCC1101 in Rats

The acute oral toxicity study of BS IDCC 1101 was performed at Korea Testing & Research Institute (Hwasun-gun, Jeollanam-do, Korea), following the guidelines of organization for economic cooperation and development (OECD) for testing chemicals (No. TGK-2021-000125, Figure S1). The female Sprague–Dawley (SD) rats were bred under the following environmental conditions: 21.1–22.3 °C, 40.5–58.0% relative humidity, air exchange (10–20) times/h, 12 h light/dark cycle, illumination 150–300 lux, 270 × 500 × 200 mm (W × D × H) cage size, and fewer than three rats per cage. Following adaptation for a week, 12 healthy rats (three rats per group, 9 and 10 weeks of age) were randomly divided into four groups. The rats were allowed access to food and water. After 16-h fasting, BS IDCC1101 (1.03 × 10^11^ CFU/g in distilled saline solution) was orally administered to rats at concentrations of 300 and 2000 mg/kg body weight. The rats in the control group were administered saline solution without bacterial strains. The body weight changes and clinical signs were carefully observed for 14 days. At the end of the period, all rats were fasted for 12 h and anesthetized using CO_2_. Necropsy and the histological evaluations were performed on all the rats by visual inspection and a board-certified toxicological pathologist, respectively.

## 3. Results and Discussion

### 3.1. Genomic Characteristics and Taxonomic Classification of BS IDCC1101

**Figure 1:**
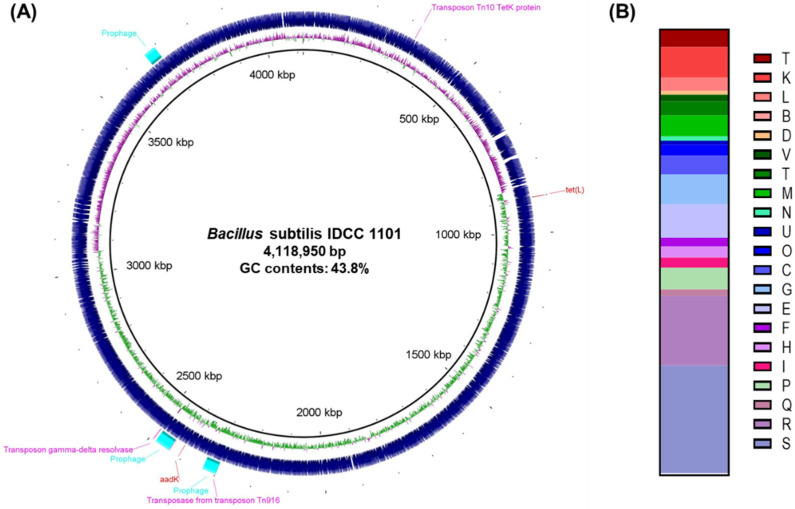
(**A**) Genome map and (**B**) annotated functional genes of *B*. *subtilis* IDCC1101. The ORFs were grouped based on the EggNOG standard into translation, ribosomal structure, and biogenesis (J); transcription (K); replication, recombination, and repair (L); chromatin structure and dynamics (B); cell cycle control, cell division, and chromosome partitioning (D); defense mechanisms (V); signal transduction mechanisms (T); cell wall, membrane, and envelope biogenesis (M); cell motility (N); intracellular trafficking, secretion, and vesicular transport (U); posttranslational modification, protein turnover, and chaperones (O); energy production and conversion (C); carbohydrate transport and metabolism (G); amino acid transport and metabolism (E); nucleotide transport and metabolism (F); coenzyme transport and metabolism (H); lipid transport and metabolism (I); inorganic ion transport and metabolism (P); secondary metabolite biosynthesis, transport, and catabolism (Q); general function prediction only (R); and function unknown (S).

BS IDCC1101 had a single chromosomal DNA without the plasmid DNA, consisting of 4,118,950 bp with GC contents of 43.8% (Figure 1A). Among the 4056 ORFs in the genome, 3077 ORFs were confirmed as functional genes. The greatest EggNOG functional group involved in the general function prediction was only 15.7%, followed by transcription (6.8%), carbohydrate transport and metabolism (6.8%), inorganic ion transport and metabolism (4.9%), cell wall/membrane/envelope biogenesis (4.6%), and energy production and conversion (4.2%) (Figure 1B). Interestingly, the annotated coenzyme transport and metabolism (E) group of BC IDCC1101 harbored gene clusters encoding vitamins such as B_1_ (thiomine, *thiC*, *thiE*, *thiF*, *thiG*, *thiL*, *thiN*, *thiS*, and moeB), B_2_ (riboflavin, *ribBA*, *ribD*, *ribE*, *ribF*, and *ribH*), B_7_ (Biotin, *bioA*, *bioB*, *bioD*, *bioF*, and *bioW*), B_9_ (foltate, *folB*, *folC*, *folE*, *folK*, and *folP*), and K_2_ (menaquinone, *menA*, *menD*, and *dxs*). *B*. *subtilis* is used as a microbial cell factory for the industrial production of vitamins, such as B_2_ produced by *B*. *subtilis* 125 [39] and K_2_ produced by *B*. *subtilis* 20-QT [40]. Thus, the multiple gene clusters involved in the vitamin synthesis of BS IDCC1101 may provide benefits as a probiotic.

According to the QPS, taxonomical identification of probiotics at the species level is the first step to confirming the safety of bacterial diets, providing information about their metabolic and genomic characteristics and growth conditions for the manufacturing process [41]. The species boundary based on ANI values is generally recognized as above 95% [42]. BS IDCC1101 was confirmed as a *B*. *subtilis* strain due to its high ANI values of 99.1% with BS subsp. *subtilis* strains 168 (type strain for BS) and 99.6% with BS SRCM 103971. In the BLASTn analysis (Table S1), BS IDCC1101 also had ~99% sequence identity to BS SRCM 103971, BS SRCM 103551, and BS SRCM 103696, which were isolated from traditional Korean fermented foods, including soy sauce (Ganjang), red pepper paste (Gochujang), soybean paste (Doenjang), and salted seafood (Jeotgal) [43]. 

**Figure 2:**
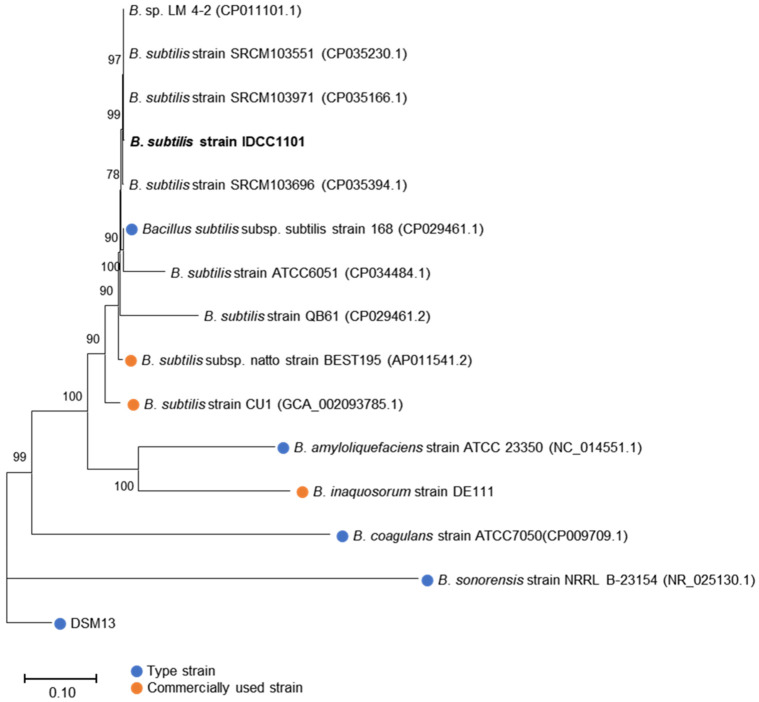
Phylogenetic tree of *B*. *subtilis* IDCC 1101 and 15 other *Bacillus* strains based on the concatenated housekeeping genes, including *groEL*, *gyrA*, *polC*, *purH*, *rpoB*, and 16S rRNA. The blue circles indicate bacterial type strains.

The BS IDCC1101 genome was further compared with that of 15 other *Bacillus* spp. available in the NCBI database (10 October 2022) to confirm its taxonomic classification. Eight genetically similar strains from the NCBI database, three industrial *Bacillus* strains used in probiotic supplements or food, and each type strain of probiotic *Bacillus* spp. including *B*. *subtilis*, *B*. *sonorensis*, *B*. *coagulans*, and *B*. *amyloliquefaciens* were used as the *Bacillus* strains for phylogenetic analysis. The phylogenetic tree revealed that BS IDCC1101 was clustered in the same branch with nine *B*. *subtilis* strains, including type strain 168 (Figure 2). Furthermore, BS IDCC1101 exhibited a homologous relationship with *B*. *subtilis* strain BEST195 and strain CU1, indicating a close and taxonomically similar relationship to the industrial probiotic *B*. *subtilis* strains. Phylogenetic, ANI, and BLAST analyses produced congruent results, thereby taxonomically placing BS IDCC1101 within the *B*. *subtilis* group. These findings indicate that BS IDCC1101 represents a new, authentic *B*. *subtilis* strain.

### 3.2. In Silico Identification of Secondary Metabolites in the BS IDCC1101 Genome

An analysis employing the BAGEL4 database showed that the BS IDCC1101 harbored two bacteriocin clusters, sporulation killing factor and subtilosin (Figure S2) aligned with the antiSMASH analysis (Table 2). These two genes have ABC transporter ATP binding proteins as their core protein and multiple ORFs. BS IDCC1101 exhibited sporulation killing factor and subtilosin A with 100% similarity. Bacteriocins are defined as ribosomally synthesized antimicrobial peptide molecules secreted by bacteria, which are commonly employed as food preservatives due to their antibacterial activity [44]. In addition, the BS IDCC1101 genome was in silico screened for the gene clusters involved in synthesizing secondary metabolites (Table 2). The in silico analysis of the secondary metabolites revealed that the BC IDCC1101 genome has 12 biosynthetic gene clusters, seven of which were predicted as responsible for the synthesis of functional secondary metabolites (Table 2). BS IDCC1101 encodes four non-ribosomal peptide synthase (NRPS) gene clusters, including lipoheptapeptide surfactin, iron-chelating siderophore bacillibactin, dipeptide antibiotic bacilysin, and lipopeptide fengycin, as well as one hybrid polyketide synthases (PKS)/NRPS gene cluster including bacillaene and two bacteriocins. Among them, two genes encoding antibiotics, bacillaene and bacilysin, that inhibit foodborne pathogens were found in the genome [45,46]. Surfactin, one of the most investigated biosurfactants, has antibacterial activity based on the penetration of bacterial phospholipid bilayer membranes [47]. BS IDCC1101 also encodes a gene cluster related to fengycin, which is already used as a commercial biocontrol agent in agriculture due to its antifungal and antibacterial activity [48]. For bacillibactin, a previous study reported that the genes involved in its biosynthesis are related to antibacterial and antifungal activities [49]. Thus, the presence of functional secondary metabolites in BS IDCC1101 can play a significant role in developing an industrial probiotic strain.

### 3.3. Safety Assessment of BS IDCC1101

**Figure 3:**
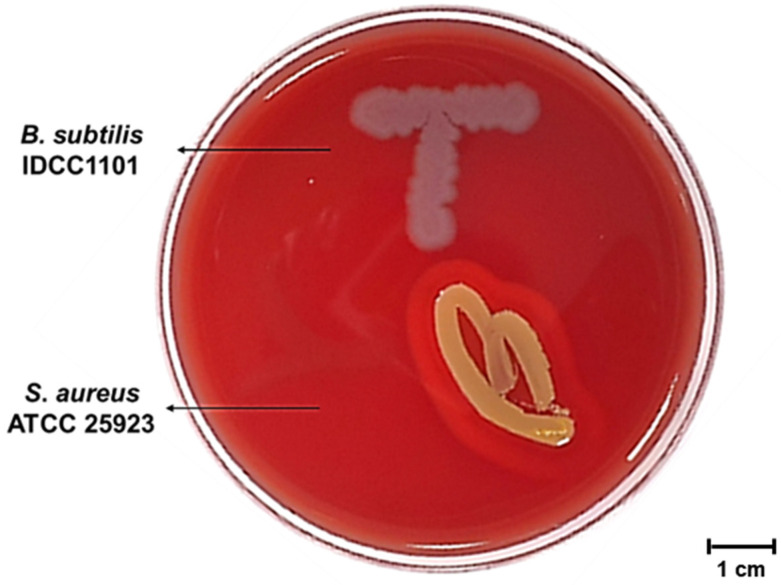
The hemolytic activity of *B*. *subtilis* IDCC1101.

To assess genomes for virulence factors (VFs), the complete sequence of BS IDCC1101 was aligned against the virulence factor database (VFDB). Among the VFs found in the *B*. *subtilis* database, 10 VFs associated with polyglutamic acid capsules, bacillibactin, and hemolysin were confirmed in the genome of BS IDCC1101 (Table 3). As mentioned earlier, the bacillibactin encoded by the *dhb* operon did have innate toxicity. It has been used to treat Parkinson’s disease due to its ability to chelate and utilize ferric iron [50]. Four VF genes, namely, *capA*, *capB*, *capC*, and *capD*, are involved in the polyglutamate (PGA) synthesis system [51]. Despite the similarity between the polymeric matrix of biofilm and the PGA in *B*. *subtilis*, a previous study reported that these genes did not participate in the biofilm formation of *B*. *subtilis* NCIB3610 [48]. Furthermore, several strains of *Bacillus,* such as *B*. *licheniformis* ATCC9945 [52], *B*. *amyloliquefaciens* LL3 [53], and *B*. *subtilis* ZJU-7 [54], were also known to produce PGA strains that were commercially used. In fact, as highly conserved genes in various *Bacillus* species [55], PGA genes are commonly found in natto and Cheonggukjang using *Bacillus* [56]. In the meantime, the *hlyIII* gene can encode a putative membrane hydrolase that induces hemolytic activity when exposed to blood [57]. However, despite the presence of *hlyIII* in the BS IDCC1101 genome, it did not exhibit hemolytic activity on the blood agar (Figure 3) as well as genes associated with hemolytic enterotoxins (*hblA*, *hblC*, and *hblD*) (Table 4). In addition, BS IDCC1101 did not carry other major enterotoxins genes produced by *Bacillus* strains (Table 4), such as non-hemolytic NHE (*nheA*, *nheB*, and *nheC*) enterotoxins, cytotoxin K (*cytK*), diarrheagenic toxins (*bceT* and *entFM*), and emetic toxin (*ces*) genes. Thus, BS IDCC1101 was confirmed to be safe for industrial use due to the absence of enterotoxin genes.

### 3.4. Hemolysis Activity of B. subtilis IDCC1101

Probiotic strains should be absent from hemolytic activity, which is the ability to lyse red blood cells with the release of intracellular material and resulting in the release of hemoglobin, for its safety [58]. Hemolysis assay can be used for rapid initial toxicity assessment. As shown in Figure 3, BS IDCC1101 did not show hemolytic activity since there was no clear zone around its colony (Figure 3), whereas *S*. *aureus* ATCC 25923, as a positive control, showed a clear zone. Similarly, several studies that reported *B*. *subtilis* for industrial use did not exhibit hemolytic activities on blood agar, including strains of CU1 [59], natto [60], and VKPM B2335 [61] (Table S2). Hemolysis can be categorized into three types: β-hemolysis, causing complete lysis of the red blood cells; α-hemolysis, forming green colonies due to methemoglobin; and γ-hemolysis, causing no damage to hemoglobin [62]. γ-hemolysis can be administered safely because it indicates hemolysis incompetency in the host body [8,58]. Interestingly, although the genome of BS IDCC1101 contained a gene encoding putative membrane hydrolase (*hlyIII)* (Table 3), it showed γ-hemolysis. In conclusion, BS IDCC1101 is safe concerning hemolytic activity.

### 3.5. Genotypic and Phenotypic Antibiotic Susceptibility of BS IDCC1101

Two genes, *aadK* and *tet(L*), were identified in the analysis of the antibiotic-resistant genes (Table 5) with 98.8 and 98.7% identities, respectively. This finding implied the possibility of resistance against streptomycin and tetracycline. From the phenotypic analysis (Table 6), BS IDCC1101 was susceptible to all antibiotics except for streptomycin, which agreed with other studies using BS 50 [63], MB40 [64], VKPM B2335 [61], and KATMIRA1933 [65] (Table S2). Streptomycin resistance is common and widespread throughout *Bacillus* spp. and is considered an intrinsic genetic property [66]. The additional findings of three incomplete prophage regions (data were not shown) and four transposons in BS IDCC1101 (Table 7) suggested the horizontal transfer of antibiotic resistance genes [53]. However, the locations of *aadK* and *tet(L*) genes were not in the mobile genetic elements (Figure 1A), so these genes may not have horizontally transferred into other bacteria and/or human gut microbiota. Overall, genotypic and phenotypic analyses validated the safety of BS IDCC1101 as an industrial probiotic.

### 3.6. Enzyme Activities and Carbohydrate Utilization of BS IDCC1101

BS IDCC1101 exhibited enzyme activities related to carbohydrate metabolism (α-galactosidase, β-galactosidase, and α-glucosidase, β-glucosidase), lipid metabolism (esterase and esterase lipase), phosphate metabolism (alkaline phosphatase and Naphthol-AS-BI-phosphohydrolase), and vitamin metabolism (acid phosphatase) in the enzymatic activity assay (Table 8). Most importantly, β-glucuronidase, which increases the risk of colorectal cancer as a carcinogenic compound [67], was not found in BS IDCC1101. 

API 50 CHL/CHB identification was used to characterize the strain and establish a carbohydrate utilization profile. BS IDCC1101 fermented 25 carbohydrates, including glycerol, L-arabinose, ribose, D-xylose, galactose, D-glucose, D-fructose, inositol, mannitol, α-methyl-D-mannoside, α-methyl-D-glucoside, N-acetyl-glucosamine, amygdaline, arbutine, esculine, salicine, cellobiose, lactose, melibiose, sucrose, trehalose, melizitose, D-raffinose, amidon, and xylitol (Table 9). Additionally, lactose gentiobiose and L-arabitol were weakly utilized. High carbohydrate fermentation activity is considered proof of strong viability [38], further supporting the use of BS IDCC1101 as an industrial probiotic.

### 3.7. Production of Biogenic Amine and Lactate by BS IDCC1101

BAs are basic nitrogenous compounds primarily generated by the microbial decarboxylation of amino acids [68]. These molecules can cause adverse health effects in humans, including hypertension, headache, nausea, diarrhea, and even death [69]. Owing to microorganisms’ highly diverse abilities to produce BAs in foods [70], BA production was evaluated as a safety criterion. BS IDCC1101 did not produce any known health-threatening BAs, such as tyramine, histamine, putrescine, 2-phenethylamine, cadaverine, or tryptamine (Table 10). Although the phenotypic expression may vary depending on the environmental conditions [71], the BA production analysis results for BS IDCC1101 may suggest that it is potentially safe in terms of BA production, constant with previous studies [72,73].

The proportion of D-/L-lactate in BS IDCC1101 was determined to evaluate its safety. BS IDCC1101 did not produce D-lactate or L-lactate (Table 10). Lactate, a metabolic product of pyruvate following anaerobic glycolysis, exists as two enantiomers, the L and D forms, owing to its asymmetric carbon atoms [74]. In humans, D-lactate accumulation in the blood may cause health problems, such as D-lactate acidosis and neurotoxic effects, due to its poor capacity to metabolize the D-lactate [75]. The D-lactate production by BS IDCC1101 was below the detection level, indicating that BS IDCC1101 could be used for industrial applications.

### 3.8. Cytotoxicity of BS IDCC1101

**Figure 4:**
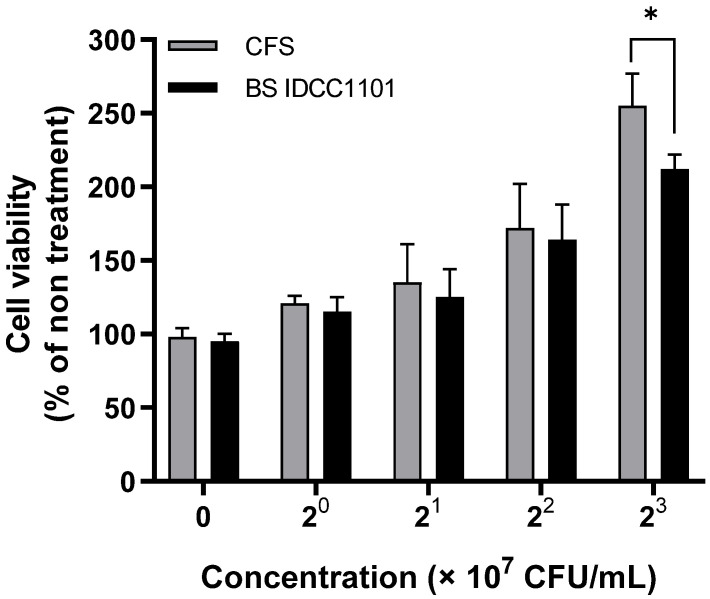
The cytotoxic effect of *B*. *subtilis* IDCC1101 on the viability of the HaCaT cell. The data are expressed as mean ± standard deviation across triplicate technical replicates. The symbol (*) indicates significantly different between whole cell and CFS of BS IDCC1101 at *p* < 0.05.

The HaCaT cell was treated with CFS of BS IDCC1101 to determine its cytotoxic effect on cell viability. Both whole cells of BS IDCC1101 and its supernatant did not affect the viability of HaCaT cells (>100% viability) at all concentrations (Figure 4). These results agreed with the non-toxic effect of BS50 on Caco-2 cells [63], BS CU1 on Vero cells [59], BS natto on HT29-16E cells, and BS PY79 on GEp-2 and Caco-2 cells [76] (Table S2). Thus, it was concluded that BS IDCC1101 was a non-toxigenic strain and, therefore, could be used as a probiotic strain.

### 3.9. Acute Oral Toxicity of BS IDCC1101 in Rats

The acute oral toxicity of BS IDCC1101 was investigated using a single-dose acute oral toxicity method. The oral administration of BS IDCC1101 in SD female rats did not cause significant changes in body weight in rats at doses of 300 and 2000 mg/kg B.W. (Table 11). Furthermore, all rats had no mortality, gross pathological changes, or abnormal necropsy findings for 14 consecutive days (Tables S3 and S4). These results indicate that BS IDCC1101 did not negatively affect the health of the rats. The acute oral toxicity evaluation of other *B. Subtilis* strains, including *B. subtilis* MB40 in rats [64], *B. subtilis* natto in pigs and rabbits [13], and *B. subtilis* VKPM B2335 in BALB/c mice [61], showed similar non-toxic findings (Table S2). Therefore, it can be concluded that BS IDCC1101 does not exhibit toxicity in rats.

## 4. Conclusions

The safety of BS IDCC1101 was evaluated via both genomic and phenotypic techniques. The in silico investigation of the BS IDCC1101 genome and cell cytotoxicity analysis revealed that BS IDCC1101 did not exhibit any substantial safety concerns, such as toxicity toward humans and transfer of antibiotic resistance or damage to gut microbiota. In addition, phenotypic analyses indicated that the strain did not produce toxic substances. Furthermore, BS IDCC1101 exhibited broader enzyme activity and carbohydrate utilization without posing safety concerns. BS IDCC1101 did not demonstrate acute oral toxicity in female rats. Based on these findings and the long history of safe consumption of *B. subtilis* in traditional fermented soybean foods, we can conclude that BS IDCC1101 is potentially safe. Thus, it is concluded that BS IDCC1101 meets the requirements of the Food and Agriculture Organization (FAO) and World Health Organization (WHO) guidelines. A clinical trial of human tolerability will be conducted to support the safe use of BS IDCC1101 for industrial applications.

## Figures and Tables

**Table 1:** Sequences of primer sets used in this study.

Target Gene	Primer Sequence (5’–3’)		Product (bp)	Reference
	Forward	Reverse		
*hblA*	AAG CAA TGG AAT ACA ATG GG	AGA ATC TAA ATC ATG CCA CTG C	1154	[33]
*hblC*	GAT ACY AAT GTG GCA ACT GC	TTG AGA CTG CTC GYT AGT TG	740	[33]
*hblD*	ACC GGT AAC ACT ATT CAT GC	GAG TCC ATA TGC TTA GAT GC	582	[33]
*nheA*	TAC GCT AAG GAG GGG CA	GTT TTT ATT GCT TCA TCG GCT	499	[33]
*nheB*	CTA TCA GCA CTT ATG GCA G	ACT CCT AGC GGT GTT CC	769	[33]
*nheC*	CGG TAG TGA TTG CTG GG	CAG CAT TCG TAC TTG CCA A	581	[33]
*cytK*	GTA ACT TTC AAT GAT GAT CC	GAA TAC TAA ATA ATT GGT TTC C	505	[32]
*bceT*	CGT ATC GGT CGT TCA CTC GG	TTT CTT TCC CGC TTG CCT TT	924	[33]
*entFM*	ATG AAA AAA GTA ATT TGC AGG	TTA GTA TGC TTT TGT GTA ACC	1269	[32]
*ces*	GGT GAC ACA TTA TCA TAT AAG GTG	GTA AGC GAA CCT GTC TGT AAC AAC A	1271	[32]

**Table 2:** Identified biosynthetic gene clusters of secondary metabolites in *B*. *subtilis* IDCC1101 using antiSMASH.

Cluster Type *	Most Similar Cluster (Accession Number)	Location (nt)	Identity (%)	Functions
NRPS	Surfactin (AJ575642.1)	450,863–514,298	82	Antibacterial
RiPP	Sporulation killing factor (AL009126.3)	644,154–666,225	100	Antibacterial, inhibition of sporulating
NRPS	Bacilysin (CP000560.1)	1,196,654–238,072	100	Antibacterial, antifungal
RiPP	Subtilosin A (AJ430547.1)	1,241,370–1,262,981	100	Antibacterial, hemolytic activity
CDPS	–	1,477,277–1,498,023	–	–
NRPS	Bacillibactin (AL009126.3)	1,783,015–1,829,009	100	Antibacterial, antifungal
NRPS-like	Capsular polysaccharide (MH190222.1)	2,340,772–2,382,008	10	–
T3PKS	–	2,804,413–2,845,306	–	–
Terpene	–	2,894,249–2,915,104	–	–
NRPS	Fengycin (CP000560.1)	2,991,749–3,068,802	100	Antibacterial, antifungal
NRPS/PKS	Bacillaene (AJ634060.2)	3,141,488–3,246,742	100	Antibacterial, inhibition of sporulation
Terpene	–	3,836,536–3,857,339	–	–

* NRPS: non-ribosomal peptide synthases; RiPP: ribosomal synthesized and post-translationally modified peptide; CDPS: tRNA-dependent cyclodipeptide synthases; T3PKS: type III polyketide synthases; PKS: polyketide synthases.

**Table 3:** Genome screening of *B*. *subtilis* IDCC1101 for detecting virulence factors using the VFDB database.

Virulence Factor	Gene	Organisms	Accession	Identity (%)
Polyglutamic acid capsule	*capA*	*B*. *subtilis* subsp. s*ubtilis* str. 168	NP_391469	99.7
Polyglutamic acid capsule	*capB*	*B*. *subtilis* subsp. *subtilis* str. 168	NP_391471	99.7
Polyglutamic acid capsule	*capC*	*B*. *subtilis* subsp. *subtilis* str. 168	NP_391470	100.0
Polyglutamic acid capsule	*capD*	*B*. *subtilis* subsp. *subtilis* str. 168	NP_389723	99.2
Bacillibactin	*dhbA*	*B*. *subtilis* subsp. *subtilis* str. 168	NP_391080.2	100.0
Bacillibactin	*dhbB*	*B*. *subtilis* subsp. *subtilis* str. 168	NP_391077.1	100.0
Bacillibactin	*dhbC*	*B*. *subtilis* subsp. *subtilis* str. 168	NP_391079.2	99.0
Bacillibactin	*dhbE*	*B*. *subtilis* subsp. *subtilis* str. 168	NP_832067.1	99.3
Bacillibactin	*dhbF*	*B*. *subtilis* subsp. *subtilis* str. 168	NP_391076.3	100.0
Hemolysin III	*hlyIII*	*B*. *subtilis* subsp. *subtilis* str. 168	NP_390062	99.53

**Table 4:** Detection of enterotoxin genes in *B*. *subtilis* IDCC1101.

Bacteria	Enterotoxin Gene									
	*hblA*	*hblC*	*hblD*	*nheA*	*nheB*	*nheC*	*cytK*	*bceT*	*entFM*	*ces*
*B*. *cereus* ATCC 14579^T^	+ ^a^	+	+	+	+	+	+	+	+	−
*B*. *subtilis* IDCC1101	− ^b^	−	−	−	−	−	−	−	−	−

^a^ + PCR product of the expected size was seen. ^b^ − No PCR product was formed.

**Table 5:** Antibiotic resistance genes detected in *B*. *subtilis* IDCC1101 using ResFinder.

Resistance Gene	Antibiotic	Location (nt)	Accession	Identity (%)	E-Value
*aadK*	Streptomysin	2,407,895–2,408,747	M26879	98.8	0
*tet(L)*	Tetracycline	898,343–899,719	X08034	98.7	0

**Table 6:** Minimum inhibitory concentration and antibiotic susceptibility of *B*. *subtilis* IDCC1101.

Class	Antibiotic	MIC (µg/mL)		Assessment ^b^
		Cutoff Values (µg/mL)	*B*. *subtilis* IDCC1101	
Aminopenicillins	Ampicillin	− ^a^	<0.125	–
Glycopeptides	Vancomycin	4	<0.125–0.25	S
Aminoglycosides	Gentamicin	4	2–4	S
Aminoglycosides	Kanamycin	8	4	S
Aminoglycosides	Streptomycin	8	64	R
Macrolides	Erythromycin	4	<0.125	S
Lincosamides	Clindamycin	4	1–2	S
Tetracyclines	Tetracycline	8	1–2	S
Amphenicols	Chloramphenicol	8	2	S

^a^ −, Cutoff value is not established in the EFSA guidelines (2018). ^b^ Assessment of antibiotic susceptibility. S: susceptible; R: resistance.

**Table 7:** Transposases and transposons predicted in the *B*. *subtilis* IDCC1101 genome.

Gene	Location (nt)	Strand
Transposon Tn10 TetD protein	327,982–328,854	+
Transposase from transposon Tn916	2,298,050–2,299,207	−
Tetracycline repressor protein class B from transposon Tn10	2,436,909–2,437,589	+
Transposon gamma-delta resolvase	2,488,579–2,490,081	+

**Table 8:** Enzyme activities of *B. subtilis* IDCC1101.

Enzyme	Activity	Enzyme	Activity
Alkaline phosphatase	+ ^a^	Naphthol-AS-BI-phosphohydrolase	+
Esterase	+	α-galactosidase	+
Esterase lipase	+	β-galactosidase	+
Acid phosphatase	+	α-glucosidase	+
Lipase	− ^b^	β-glucosidase	+
Leucine arylamidase	−	β- glucuronidase	−
Valine arylamidase	−	N-acetyl-β-glucosaminidase	−
Cystine arylamidase	−	α-mannosidase	−
Trypsin	−	α-fucosidase	−
α-chymotrypsin	−		

^a^ + Enzyme activity. ^b^ − No enzyme activity.

**Table 9:** Carbohydrate fermentation activities of *B*. *subtilis* IDCC1101.

Substrate	Result ^a^	Substrate	Result	Substrate	Result
Glycerol	+	Mannitol	+	D-Raffinose	+
Erythritol	−	Sorbitol	−	Amidon	+
D-Arabinose	−	α-Methyl-D-mannoside	+	Glycogen	−
L-Arabinose	+	α-Methyl-D-glucoside	+	Xylitol	+
Ribose	+	N-Acetyl-Glucosamine	+	Gentibiose	+
D-Xylose	+	Amygdaline	+	D-Turanose	−
L-Xylose	−	Arbutine	+	D-Lyxose	−
Adonitol	−	Esculine	+	D-Tagatose	−
β-Methyl-xylose	−	Salicine	+	D-Fucose	−
Galactose	+	Cellobiose	+	L-Fucose	−
D-Glucose	+	Maltose	−	D-Arabitol	−
D-Fructose	+	Lactose	+	L-Arabitol	+
D-Mannose	−	Melibiose	+	Gluconate	−
L-Sorbose	−	Sucrose	+	2-keto-gluconate	−
Rhamnose	−	Trehalose	+	5-keto-gluconate	−
Dulcitol	−	Inuline	−		
Inositol	+	Melizitose	+		

^a^ + Carbohydrate utilization; − no carbohydrate utilization.

**Table 10:** Biogenic amine concentration and L-/D-lactate proportions in CFS of *B*. *subtilis* IDCC1101.

Biogenic Amine (mM)	Result
Tyramine	ND ^a^
Histamine	ND
Putrescine	ND
2-Phenethylamine	ND
Cadaverine	ND
Tryptamine	ND
**D-/L- lactate proportion**	**Result**
L-lactate (g/l)	ND
D-lactate (g/L)	ND
L-form (%)	ND
D-form (%)	ND

^a^ ND: not detected.

**Table 11:** Body weight changes in rats administered *B*. *subtilis* IDCC1101.

Group	Dose (g/kg BW ^1^)	Day after Administration				
		0	1	3	7	14
9 weeks old	300	216.2 ± 9.0	240.3 ± 8.9	251.3 ± 13.7	257.1 ± 16.2	269.7 ± 16.1
	2000	205.5 ± 4.2	232.6 ± 2.7	237.3 ± 2.6	246.5 ± 4.3	254.2 ± 7.1
10 weeks old	300	231.8 ± 9.6	257.5 ± 13.8	254.6 ± 14.3	261.2 ± 16.8	266.6 ± 12.1
	2000	220.4 ± 3.9	246.0 ± 4.2	256.2 ± 6.4	263.7 ± 4.3	266.2 ± 6.1

^1^ BW, body weight.

## Data Availability

Not applicable.

## Supplementary Materials

The following supporting information can be downloaded at: https://www.mdpi.com/article/10.3390/microorganisms10122494/s1, Figure S1: The GLP and quality assurance statements for acute oral toxicity study of BS IDCC1101.; Figure S2: Organization of bacteriocin cluster genes.; Table S1: Summary of BLASTn screening results of *B*. *subtilis* IDCC1101.; Table S2: Biochemical properties of *Bacillus subtilis* reported for use as a commercial probiotic.; Table S3: Mortality and Clinical signs of female rats.; Table S4: Necropsy findings of female rats.
