# Supplementary material for: Safety Evaluation of Bacillus subtilis IDCC1101, Newly Isolated from Cheonggukjang, for Industrial Applications
Source: Microorganisms. 2022 Dec 16;10(12):2494. doi: 10.3390/microorganisms10122494 (PMC9784242; doi:10.3390/microorganisms10122494)
Supplement: Supplementary file 1 [file microorganisms-10-02494-s001.zip › microorganisms-2070557-supplementary.pdf]

Supplementary data

GLP Statement

Study title : Acute oral toxicity study of *Bacillus subtilis* IDCC 1101 in rat

Study number : TGK-2021-000125

Sponsor

Name : ILDONG BIOSCIENCE Co., LTD
Address : 17, Poseunggongdan-ro, Poseung-eup, Pyeongtaek-si, Gyeonggi-do, Republic of Korea
Representative : LEE JANG HYEY
Person in charge : Yo Hwan Kim
Department and position : Quality Control Team Manager
Contact Number : Tel. +82-31-646-3148 Fax. 070-7500-2592

Test facility

Name : Korea Testing and Research Institute, Hwasun
Address : 12-63, Sandan-gil, Hwasun-eup, Hwasun-gun, Jeollanam-do, Republic of Korea
Test facility management : Park Myeong-kyu
Contact Number : Tel. +82-61-370-7700 Fax. +82-61-370-7777

This study was conducted under the supervision of the study director and is in compliance with the principles of Good Laboratory Practice.
This study was conducted under the supervision of the study director and is in compliance with the principles of Good Laboratory Practice.

1. Good Laboratory Practice
1.1. OECD "Principles of Good Laboratory Practice" ENV/MC/CHEM (98)17 (as revised in 1997)
2. Test regulation
2.1. OECD Guideline for testing of chemicals, Section 4, TG 423 "Acute Oral Toxicity-Acute Toxic Class Method" (Adopted : 17th December 2001)

Study director (Signed in the original report, annex 5) Jang Seong-yong, M.S. — Date

Test facility management (Signed in the original report, annex 5) Hong Seung-guk, M.S. — Date

× *This report was translated a Korean sentence into English according to sponsor' request by study director. The original data had not been changed.*

Translator Jang Seong-yong (signature) Jang Seong-yong, M.S. — 2021-06-04 Date

- I -

Quality Assurance Statement

Study title : Acute oral toxicity study of *Bacillus subtilis* IDCC 1101 in rat

Study title : TGK-2021-000125

Study Phases	Inspections	Reports to study director and Test facility management
Draft study plan audit	2021-03-25	2021-03-25
Study plan audit	2021-03-26	2021-03-26
Animal receipt audit	2021-03-30	2021-03-30
Body weight audit	2021-03-30	2021-03-30
Group assignment audit	2021-04-05	2021-04-05
Preparation of test substance audit (1)	2021-04-06	2021-04-06
Preparation of test substance audit (2)	2021-04-14	2021-04-14
Test substance administration audit	2021-04-06	2021-04-06
Clinical signs audit (1)	2021-04-06	2021-04-06
Necropsy audit (1)	2021-04-20	2021-04-20
Necropsy audit (2)	2021-05-14	2021-05-14
Raw data audit	2021-05-25	2021-05-25
Draft final report audit	2021-05-25	2021-05-25
Final report audit	2021-05-27	2021-05-27

Inspections of the routine and repetitive procedures that constitute the study was carried out as a continuous process designed to encompass the major phases at or about the time this study was in progress.
This report has been audited by Quality Assurance Unit of Korea Testing and Research Institute, Hwasun and is considered to be an accurate account of the raw data generated and of the procedures followed.
Inspections were accomplished as noted, and reported to the study director and management immediately following their completion. Based on these inspections and the review of the report, this study was conducted and reported in conformance with the Good Laboratory Practice regulations.

Quality assurance personnel (Signed in the original report, annex 6) Seo Mu-yeb, M.S. — Date

- II -

Figure S1. The GLP and quality assurance statements for acute oral toxicity study of BS IDCC1101.

Table S1. Summary of BLASTn screening results of *B. subtilis* IDCC1101.

Description	Accession	Coverage	E-value	Identities (%)
Bacillus subtilis strain SRCM103971 chromosome, complete genome	CP035166.1	34.35	0.0	99
Bacillus sp. LM 4-2, complete genome	CP011101.1	34.7	0.0	99
Bacillus subtilis strain SRCM103551 chromosome, complete genome	CP035230.1	12.96	0.0	99
Bacillus subtilis strain QB61 chromosome, complete genome	CP029461.2	11.21	0.0	99
Bacillus subtilis strain SRCM103696 chromosome, complete genome	CP035394.1	11.07	0.0	99

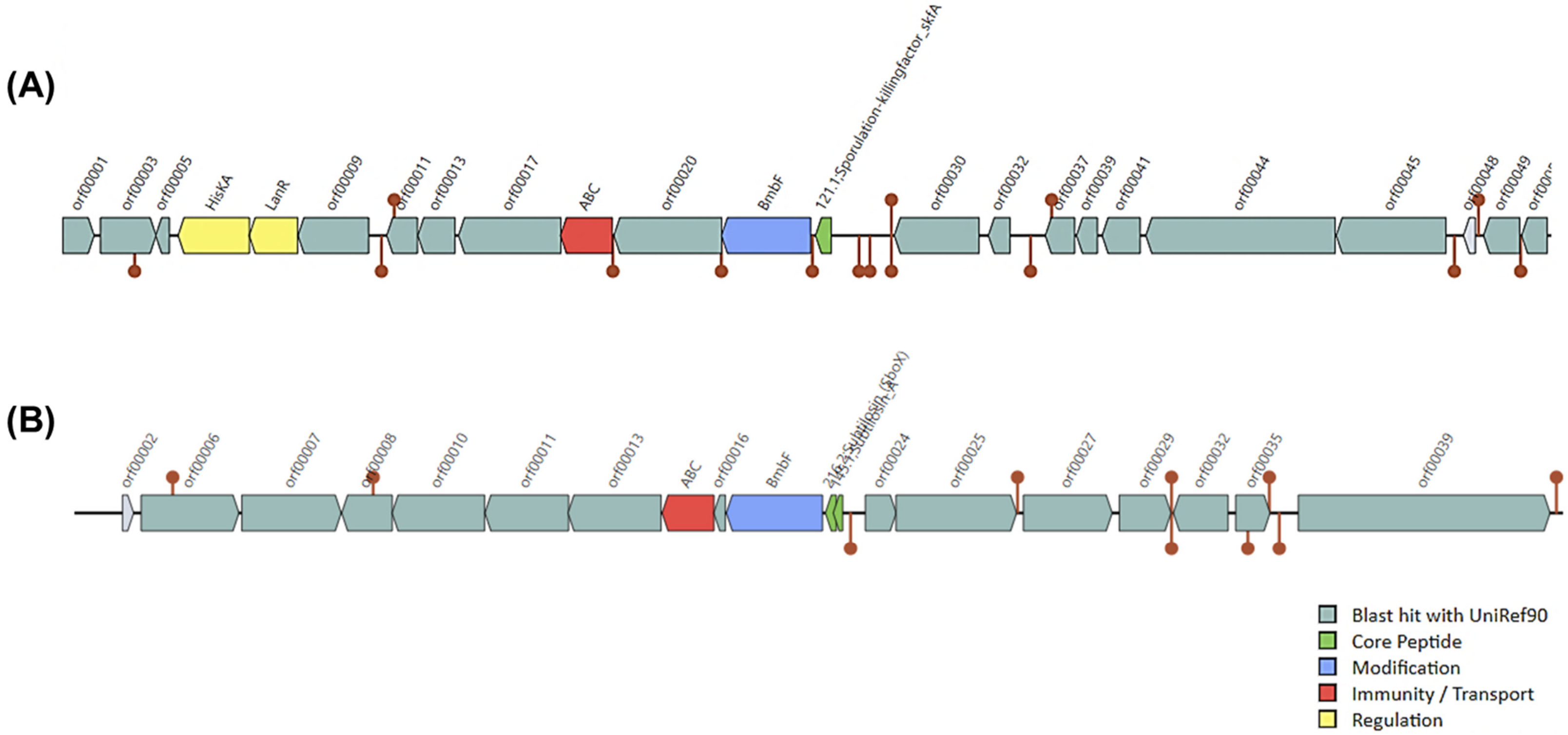

Figure S2. Organization of bacteriocin cluster genes.

Table S2. Biochemical properties of *Bacillus subtilis* reported for use as a commercial probiotic

B. subtilis	Source	Antibiotic resistance (MIC μg/mL)	Enzyme activity	L-/D-lactate	BA[1)]	Hemolytic activity	Cell cytotoxicity		Acute oral toxicity	Ref.
							Cell	Cytotoxicity		
BS IDCC1101	Cheonggukjang	Streptomycin (64)	ND	ND	ND	γ	HaCaT	Non-toxic	Non-adverse	
BS50	Soil	Streptomycin (125)	-[2)]	-	-	α	Caco-2	Non-toxic	-	[1]
CU1	Soil/water	ND	-	-	-	γ	Vero	Non-toxic	-	[2]
MB40	Soil	Streptomycin (>32)	-	-	-	-	-	-	Non-adverse	[3]
Natto	Natto	ND	-	-	-	γ	HT29-16E	Non-toxic	Non-adverse	[4]
PY79	Straw	-	-	-	-	-	HEp-2, Caco-2	Non-toxic	-	[5]
VKPM B2335	Straw	Oxacillin*	-	-	-	γ	Caco-2 and mucin	Low adhesion	Non-adverse	[6]
KATMIRA1933	Yogu Farm	Streptomycin, bacitracin*	-	-	-	β	-	-	-	[7]

1) BA: biogenic amine.

2) -: not tested; ND: not detected.

* MIC was not investigated since they used paper disc method.

Table S3. Mortality and Clinical signs of female rats.

Group	Dose (mg/kg B.W.)	Mortality	Animal number	Hours after administration					Days after administration													
				0.5	1	2	3	4	1	2	3	4	5	6	7	8	9	10	11	12	13	14
			2101	N	N	N	N	N	N	N	N	N	N	N	N	N	N	N	N	N	N	N
G1	300	0% (0/3)[a]	2102	N	N	N	N	N	N	N	N	N	N	N	N	N	N	N	N	N	N	N
			2103	N	N	N	N	N	N	N	N	N	N	N	N	N	N	N	N	N	N	N
			2201	N	N	N	N	N	N	N	N	N	N	N	N	N	N	N	N	N	N	N
G2	300	0% (0/3)	2202	N	N	N	N	N	N	N	N	N	N	N	N	N	N	N	N	N	N	N
			2203	N	N	N	N	N	N	N	N	N	N	N	N	N	N	N	N	N	N	N
			2301	N	N	N	N	N	N	N	N	N	N	N	N	N	N	N	N	N	N	N
G3	2000	0% (0/3)	2302	N	N	N	N	N	N	N	N	N	N	N	N	N	N	N	N	N	N	N
			2303	N	N	N	N	N	N	N	N	N	N	N	N	N	N	N	N	N	N	N
			2401	N	N	N	N	N	N	N	N	N	N	N	N	N	N	N	N	N	N	N
G4	2000	0% (0/3)	2402	N	N	N	N	N	N	N	N	N	N	N	N	N	N	N	N	N	N	N
			2403	N	N	N	N	N	N	N	N	N	N	N	N	N	N	N	N	N	N	N

B.W.: Body weight, [a]: Number of dead animals/Number of tested animals, N: Normal

.

Table S4. Necropsy findings of female rats.

Group	Dose (mg/kg B.W.)	Animal number	Removal reason	Necropsy findings	
				External	Internal
G1	300	2101	Terminal sacrifice	No gross findings	No gross findings
		2102	Terminal sacrifice	No gross findings	No gross findings
		2103	Terminal sacrifice	No gross findings	No gross findings
G2	300	2201	Terminal sacrifice	No gross findings	No gross findings
		2202	Terminal sacrifice	No gross findings	No gross findings
		2203	Terminal sacrifice	No gross findings	No gross findings
G3	2000	2301	Terminal sacrifice	No gross findings	No gross findings
		2302	Terminal sacrifice	No gross findings	No gross findings
		2303	Terminal sacrifice	No gross findings	No gross findings
G4	2000	2401	Terminal sacrifice	No gross findings	No gross findings
		2402	Terminal sacrifice	No gross findings	No gross findings
		2403	Terminal sacrifice	No gross findings	No gross findings

B.W.: Body weight

References

1. Brutscher, L.M.; Borgmeier, C.; Garvey, S.M.; Spears, J.L. Preclinical Safety Assessment of *Bacillus subtilis* BS50 for Probiotic and Food Applications. *Microorganisms* **2022**, *10*, 1038.
2. Lefevre, M.; Racedo, S.M.; Denayrolles, M.; Ripert, G.; Desfougeres, T.; Lobach, A.R.; Simon, R.; Pélerin, F.; Jüsten, P.; Urdaci, M.C. Safety assessment of *Bacillus subtilis* CU1 for use as a probiotic in humans. *Regulatory Toxicology and Pharmacology* **2017**, *83*, 54-65.
3. Spears, J.L.; Kramer, R.; Nikiforov, A.I.; Rihner, M.O.; Lambert, E.A. Safety Assessment of *Bacillus subtilis* MB40 for Use in Foods and Dietary Supplements. *Nutrients* **2021**, *13*, 733.
4. Ruiz Sella, S.R.; Bueno, T.; de Oliveira, A.A.; Karp, S.G.; Soccol, C.R. *Bacillus subtilis* natto as a potential probiotic in animal nutrition. *Critical Reviews in Biotechnology* **2021**, *41*, 355-369.
5. Schroeder, J.W.; Simmons, L.A. Complete genome sequence of *Bacillus subtilis* strain PY79. *Genome announcements* **2013**, *1*, e01085-01013.
6. Sorokulova, I.B.; Pinchuk, I.V.; Denayrolles, M.; Osipova, I.G.; Huang, J.M.; Cutting, S.M.; Urdaci, M.C. The safety of two *Bacillus* probiotic strains for human use. *Digestive diseases and sciences* **2008**, *53*, 954-963.
7. AlGburi, A.; Volski, A.; Cugini, C.; Walsh, E.M.; Chistyakov, V.A.; Mazanko, M.S.; Bren, A.B.; Dicks, L.M.; Chikindas, M.L. Safety properties and probiotic potential of *Bacillus subtilis* KATMIRA1933 and *Bacillus amyloliquefaciens* B-1895. *Advances in Microbiology* **2016**, *6*, 432-452.
